# Digital interactions with the pharmaceutical industry: a qualitative focus group study on the perspectives of rheumatology care providers in Germany

**DOI:** 10.1186/s41927-026-00623-1

**Published:** 2026-02-11

**Authors:** Susann May, Amina Jeleskovic-Öztürk, Hannah Meisberger, Finnja Stangel, Katharina Boy, Niklas Ohm, Greta Nordmann, Robert Darkow, Johannes Knitza, Felix Muehlensiepen

**Affiliations:** 1 Center for Health Services Research, Faculty of Health Sciences Brandenburg, Brandenburg Medical School, Brandenburg Medical School Theodor Fontane, Seebad 82/83, 15562 Rüdersdorf, Brandenburg Germany; 2 Deutsches Herzzentrum der Charité – Department of Cardiology, Angiology and Intensive Care Medicine, Charitéplatz 1, 10117 Berlin, Germany; 3 Charité – Universitätsmedizin Berlin, Corporate Member of Freie Universität Berlin and Humboldt-Universität zu Berlin, Charitéplatz 1, 10117 Berlin, Germany; 4 FH Joanneum - University of Applied Sciences, Health Studies, Graz, 8020 Austria; 5 Institute for Digital Medicine, University Hospital Giessen-Marburg, Philipps University, 35037 Marburg, Germany; 6 Univ. Grenoble Alpes, CNRS, Grenoble INP, LIG Sangria, Grenoble, 38000 France

**Keywords:** Digital health, Pharmaceutical industry, Rheumatology, Digital transformation, Qualitative research

## Abstract

**Background:**

Digitalization is reshaping healthcare delivery, including how physicians and the pharmaceutical industry interact. Rheumatology care is a particularly dynamic field, where digital technologies are increasingly integrated into both clinical care and pharmaceutical engagement. Despite growing digital collaboration, little is known about healthcare professionals’ perspectives on these developments.

**Objective:**

This study explores how stakeholders in German rheumatology perceive and experience digital interactions with the pharmaceutical industry, with a focus on evolving roles, opportunities, and ethical challenges.

**Methods:**

We conducted a qualitative focus group study involving 18 stakeholders, including rheumatologists, medical assistants, and digital health professionals. Discussions focused on communication practices, collaborative models, and the integration of digital technologies. Data were analyzed using structured qualitative content analysis.

**Results:**

Participants reported a shift from in-person to digital communication with pharmaceutical representatives. While digital formats were seen as more accessible and efficient, they also led to a perceived loss of personal interaction and an increase in information overload. Digital health applications, continuing education, and clinical trials with digital endpoints were identified as key areas of collaboration. Concerns included data ownership, blurred role boundaries, and the influence of commercial interests. Participants emphasized the need for clear governance structures and patient-centered, interoperable digital solutions.

**Conclusion:**

Digitalization is altering the nature of collaboration between physicians and the pharmaceutical industry in rheumatology care. While offering new opportunities for engagement and innovation, these shifts raise ethical, structural, and practical challenges. Ensuring transparency, data protection, and role clarity will be essential to foster trust and optimize digital cooperation.

**Supplementary Information:**

The online version contains supplementary material available at 10.1186/s41927-026-00623-1.

## Background

Digitalization is fundamentally reshaping 21st-century healthcare by transforming care structures, communication, and interaction among patients, professionals, and external stakeholders. In Germany, developments such as the electronic patient record, telemedicine, and certified digital health applications (DiGA) illustrate this shift [1]. These changes extend beyond technical innovation, raising questions about ethics, care quality, and professional roles.

While older patients increasingly accept digital health technologies, challenges persist [2]. Medical professionals are expected to engage digitally in both patient care and collaborations with partners such as the pharmaceutical industry. Rheumatology care, with its high demand for ongoing care, staff shortages, and therapeutic innovation [3], exemplifies this digital transformation [4–6].

Existing research largely focuses on physician–patient interaction or specific technologies [7]. However, little is known about how digitalization affects relationships between clinicians and the pharmaceutical industry, particularly given emerging formats such as digital education, remote studies, and data-sharing via DiGA. These developments offer new opportunities but also raise ethical and regulatory questions.

Furthermore, digitalization is shifting roles: physicians become part of decentralized knowledge networks, the pharmaceutical industry acts as co-developer of care approaches, and patients assume more active roles. Despite this, empirical studies examining how physicians experience these shifts, especially in digitally advanced settings like rheumatology, remain scarce.

This study addresses that gap by exploring how rheumatology care providers perceive the digital transformation of their collaboration with the pharmaceutical industry. It aims to identify potentials and challenges, highlight shifting responsibilities, and inform future collaboration models.

## Methods

### Study design

This paper reports on an exploratory qualitative study on the stakeholder perspectives regarding digital interactions with the pharmaceutical industry in the context of rheumatology care. We conducted focus group discussions with various stakeholders, including physicians, practice staff, and other healthcare professionals.

### Ethical considerations

This study was approved by the Ethics Committee of the Brandenburg Medical School Theodor Fontane (E-02-20211028). Participants received both verbal and written information about the purpose, procedures, and significance of the study, as well as any potential benefits and risks. They had the opportunity to ask questions and were informed that participation was voluntary and that they could withdraw their consent at any time, verbally or in writing, without providing reasons. Participants were also informed that personal data would be collected and stored in a pseudonymized form, and that no data would be published that could lead to their identification.

### Recruitment

Participants were selected using purposive sampling [8]. We included stakeholders who were actively involved in rheumatology care and potentially exposed to digital interactions with the pharmaceutical industry. This included rheumatologists, other physicians, medical practice staff, digital health entrepreneurs and researchers. Participants were selected based on predefined categories to ensure a heterogeneous sample and to capture a wide range of perspectives [9]. These categories included region of practice (rural vs. urban), type of professional role, and gender. An additional inclusion criterion was an expressed interest and willingness to participate in a focus group discussion. Participants were recruited through systematic outreach via social media as well as snowball sampling.

### Data collection

A preliminary semi-structured focus group guide was developed by a multiprofessional research team. The guide included open-ended questions aimed at exploring stakeholder experiences and perceptions regarding digital interactions with the pharmaceutical industry in rheumatology care. Key topics included the use of artificial intelligence and big data in pharmaceutical research and development, stakeholder involvement in such digital projects, communication practices between healthcare professionals, digital health entrepreneurs, researchers and pharma representatives, and perceived information needs and potentials for collaboration. Additional sections addressed the integration of digital tools in clinical trials, the role of telemedicine and remote care in current practice, and ethical and legal considerations related to data use, transparency, and patient perspectives. Please refer to Supplementary Material 1 for the final interview guide.

Sociodemographic data were also collected, including profession and gender. To ensure clarity and relevance, the focus group guide was pilot-tested with five eligible participants from the target setting. All focus groups were conducted online via video conferencing between October 2022 and September 2023. Sessions were recorded and transcribed verbatim in accordance with data protection regulations. Data collection continued until meaning saturation was reached, i.e., when additional focus groups no longer yielded new meanings, perspectives, or explanatory insights relevant to the research question (rather than merely new codes) [10]. Brief field notes were taken after each session to support contextual understanding of the discussion, but were not included in the formal analysis.

### Data analysis

Qualitative analysis of the focus group discussions was conducted iteratively by members (FM, SM, KB) of the research team using Kuckartz’s structured qualitative content analysis approach [11], supported by MAXQDA Analytics Pro 2022 (version 22.1.0; Verbi GmbH). Following transcription of the audio recordings, the analysis began with a thorough reading of the transcripts, during which the material was systematically coded. In line with Kuckartz, we distinguish between codes (labels assigned to meaningful segments of text) and categories (higher-order groupings that structure the analytic framework). Categories were organized into overarching themes that guided the presentation of results. Coding followed a combined deductive-inductive strategy: some categories were derived deductively from the research questions, while others emerged inductively from the material itself. Main and subcategories were developed from the initial codes and continuously refined through consensus discussions within the research team to ensure a shared understanding of the coding framework. At the time of coding, data collection had already been completed. Two researchers independently applied the finalized coding system to the full dataset, ensuring transparency and replicability. The analysis was conducted in German. To illustrate key findings, selected quotes were translated into English by native speakers and included in the manuscript. The study was reported in accordance with the COREQ (Consolidated Criteria for Reporting Qualitative Research) guidelines (see supplementary material 2) [12].

### Reflexivity

The research team was multiprofessional and included researchers with expertise in health services research (FM, SM, RD), qualitative methods (FM, SM, KB), and digital health (FM, SM, KB, JK). Several team members have conducted previous research on digital health transformation in rheumatology (FM, SM, KB, RD, JK) and related fields. To mitigate potential preconceptions regarding digitalization and industry interactions, we used (i) a collaboratively developed and pilot-tested discussion guide, (ii) structured moderation to encourage divergent perspectives, and (iii) regular reflexive team discussions and memoing during analysis. Coding decisions were documented and discussed in consensus meetings to enhance transparency and reduce individual interpretive dominance.

## Results

A total of 18 individuals participated in the three focus groups. The participants represented a range of stakeholder groups involved in rheumatology care. The sample included rheumatologists (*n* = 11), rheumatology medical assistants (*n* = 3), an internist (*n* = 1), and digital health entrepreneurs (*n* = 3). All participating rheumatologists were male, whereas practice staff included female participants. Focus group discussions had an average duration of 72 min (range: 68–77 min). An overview of participant characteristics is provided in Table 1 to support interpretability and transferability while maintaining anonymity.

**Table 1:** Overview of the participant characteristics

Characteristic	Category	*n*
Professional role	Rheumatologist	11
	Rheumatology medical assistant (RFA)	3
	Internist	1
	Digital health entrepreneur	3
Gender	Male	14
	Female	4
Setting	Outpatient practice	6
	Hospital / outpatient clinic	4
	Other / N/A	3
Region (Germany)	Urban	12
	Rural	4
Years in practice (approx.)	0–5	3
	6–10	8
	11–20	4
	> 20	3

### Impact of digitalization on collaboration with the pharmaceutical industry in rheumatology

In the field of rheumatology care, recent developments in communication reflect a broader shift driven in part by the pharmaceutical industry. Over the past few years, pharmaceutical companies have increasingly invested in new communication strategies that go beyond traditional professional dialogues. By establishing a presence on social media and initiating patient-centered campaigns, they have contributed to the emergence of platforms that facilitate more direct and accessible interaction with affected individuals. This evolving landscape suggests that the pharmaceutical industry plays an active role in shaping how information is disseminated and how connections between patients, physicians, and companies are fostered in a more participatory and digitally-mediated environment.

Over the past three to four years, I have noticed increasing efforts in communication, with new communication channels emerging that did not previously exist or that I, as an affected individual, had not registered or perceived at the time. (Digital health entrepreneur, male)

Especially in outpatient rheumatology, personal contact with the pharmaceutical industry has noticeably declined. Due to ongoing digitalization and changing structural conditions, direct encounters have become less frequent. In their place, digital formats are increasingly used to share information. The physical presence of industry representatives has significantly decreased, while online services such as information portals and digital materials have been expanded. This has changed the nature of collaboration, shifting it toward a more distanced and formalized interaction.

Due to digitalization and the significantly reduced direct contact with the pharmaceutical industry, our interactions have become more distanced. They now provide us with more information digitally and are less physically present. (Rheumatologist, male)

Healthcare providers are increasingly recognizing the role of digital endpoints in rheumatology research, for example patient-reported outcomes (PROs) in rheumatoid arthritis, symptom tracking, and remote monitoring integrated via apps and DiGAs, particularly in clinical studies. This development has encouraged closer collaboration, for example in the area of digital health applications (DiGAs). Digital health technologies such as digital health applications enable the collection of additional clinical endpoints and are increasingly being integrated into study designs. As a result, the clinical trial landscape is evolving as more digital measurement tools are now available, validated and integrated into research processes. The pharmaceutical industry is also showing a growing interest in digital approaches, such as treatment response prediction algorithms, even though there are currently no sufficiently validated models.

Yes, there are increasingly more digital endpoints, or rather an additional Clinical Outcome Assessment, facilitated through apps and similar technologies, which are now being integrated into clinical studies. In this sense, clinical studies have also evolved, with more digital endpoints being available on the market, validated, and incorporated into research. The pharmaceutical industry is, of course, examining whether we have algorithms to predict treatment responses, which we do not yet have, at least not properly validated. (Rheumatologist, male)

The use of digital training formats has also increased. Digital education modules provided by the pharmaceutical industry are becoming an important supplement to existing offerings. While the number of in-person events has risen, participation at these physical meetings appears to be declining. In contrast, digital formats offer substantially greater reach and improved accessibility especially for healthcare professionals with limited time or those in remote locations. The industry is thus responding to changing needs in medical education and contributing to the expansion of digital learning opportunities in the outpatient setting.

Essentially, it is an addition; the use of digital training modules provided by the industry has significantly increased. For example, in-person events and other (unintelligible) events have risen exponentially, while the number of participants has rather decreased. However, accessibility and reach have increased significantly. (Rheumatologist, male)

From the perspective of healthcare providers, personal contact with pharmaceutical companies has declined, while the overall intensity of communication is perceived to have increased. Digital lectures, webinars, and other offerings have become significantly more common and are increasingly replacing physical presence. Although face-to-face interactions have become less frequent, exchanges now occur more often, for example through virtual events, invitations to online meetings, or participation in digital lectures. The pharmaceutical industry thus remains present, but in a different form, primarily through digital channels and often with a higher frequency than in the past.

Yes, that’s exactly what I meant with the lectures and offerings from the pharmaceutical company. I also have the feeling that, although we don’t see the pharmaceutical company in person, we actually have more contact with them because we are more frequently invited to online meetings or asked to speak at various events. (Internist, female)

Communication between pharmaceutical companies and startups developing Digital Health Applications, such as DiGAs, has also intensified. This exchange is now perceived as significantly easier than in the past. The pharmaceutical industry is paying closer attention to developments in the field of digital therapies, particularly regarding whether these approaches are moving beyond the proof-of-concept stage and generating robust evidence, clinical benefit, and pharmacoeconomic value. At the same time, pharmaceutical stakeholders are increasingly clear about their goals in collaborating with digital health providers. As a result, the dialogue has become more structured and effective.

We come from the digital therapies field, and we’ve noticed that the pharmaceutical industry is closely monitoring this. They are essentially observing whether the whole topic of DiGAs and digital therapies is moving out of the proof-of-concept phase and really generating evidence and added value, including pharmacoeconomic benefits. It’s slowly gaining momentum. Also, the discussions we have with pharma are becoming much easier because they are now more aware of what they actually want to achieve. (Employee of a start-up, male)

Digitalization has made communication with pharmaceutical companies more targeted and easier, in part due to the widespread use of video conferencing tools. The need to coordinate in-person meetings has decreased, making exchanges more efficient. Information can now be shared more quickly and with less effort, which many stakeholders in the outpatient sector view as a positive development. The barrier to initiating contact has been lowered, while the focus and clarity of discussions have improved.

Overall, I personally find communication with pharmaceutical companies to be more targeted and easier due to digitalization, because you can just quickly talk via Zoom now. You no longer need to arrange on-site appointments… I think it has become more efficient and easier. So, I view this rather positively. (Rheumatologist, male)

From the physicians’ perspective, communication with the pharmaceutical industry increasingly reflects the way they themselves communicate with their patients. The relationship has become more digital, more distant, and shaped more by the flow of information than by personal interaction. Physicians observe parallels in this dynamic: just as they now see patients less often in person while providing them with more digital information, the pharmaceutical industry is engaging with medical professionals in a similar way. This development is viewed with some criticism, as it suggests a form of mutual distancing through digital information overload combined with a decline in face-to-face presence.

I think if you ask us doctors, we always believe we’re at the center, that we’re in control and determine the pace and nature of digitalization. But if we’re being honest and talk about the pharmaceutical industry, they’re essentially playing the same game with us that we play with patients. Meaning, we’re distancing ourselves from patients, trying to provide them with lots of information, seeing them less frequently because things are becoming more digital. And the pharmaceutical industry is doing exactly the same with us. We see them less often in person. In the end, we’re also a kind of pawn, they keep us engaged with a sort of digital overload. To be honest, the same thing we’re doing with patients is what the pharmaceutical industry is doing with us. (Rheumatologist, male)

The pharmaceutical industry is viewed by many stakeholders as a potential partner for collaboration on various projects. Especially in light of limited funding opportunities, it is seen as a resourceful actor capable of advancing and supporting specific initiatives. Compared to statutory health insurance systems or academic research environments, the industry is sometimes perceived as taking a more pragmatic approach. This openness to collaboration is driven less by ideological alignment and more by a shared interest in moving concrete projects forward.

Well, I think when you look at projects nowadays, funding is often a major issue. In that regard, I definitely see the pharmaceutical industry as a potential partner, at the very least, they have the resources to implement and push certain topics forward. They may also take a somewhat more pragmatic approach than what we typically see in the statutory health insurance environment or in academic research contexts. (Employee of a start-up, patient with rheumatic disease, male)

Such collaboration offers outpatient clinics the opportunity to independently collect real world, practice-oriented data and to analyze it directly, beyond the framework of traditional clinical trials. Especially in a digital care context, physicians such as rheumatologists can remain the central point of contact for patients while continuously generating data streams that are automatically collected without requiring manual input. This creates the potential for closely tailored and personalized treatment adjustments based on real life care situations. At the same time, it opens new possibilities for decentralized observational studies and for building practice based registries that reflect everyday clinical reality without the need for extensive research infrastructure.

I believe that collaboration with the pharmaceutical industry especially for office-based physicians offers tremendous opportunities, particularly when care is conceived in a digital context. Assuming, and this is a hypothesis, that the treating physician remains the central point of contact for the patient, while a digital connection is maintained, then the treating physician, or rheumatologist in this case, would be in a significantly better starting position. Specialists treating chronic conditions could, more or less, maintain their own practice-based registry with tightly monitored data streams that are collected automatically, without the need for active manual input. The major opportunity arising from this is that we will, for the first time, have large datasets from patients’ everyday lives, outside the scope of clinical trials. This enables truly personalized adjustments and allows observational studies and similar research to be organized more broadly and decentrally in daily practice. Different networks of practices could initiate various studies without much effort. (Employee of a start-up, male)

For a detailed overview of the category system, please refer to supplementary material 3.

### Challenges in collaboration with the pharmaceutical industry

A key challenge in collaboration lies in the overwhelming number of digital tools made available to patients by the pharmaceutical industry. While digitalization creates new opportunities for support and engagement, the sheer volume of available tools carries the risk of confusing or overwhelming patients. Without clear structure, filtering mechanisms, or contextual guidance, patients can easily lose track, which may limit both the usability and effectiveness of these offerings.

Exactly, what comes to mind spontaneously with these inputs is the offer of many digital tools that pharmaceutical companies provide to patients. I think as a patient, you’d probably lose track of them pretty quickly. (Internist, female)

Another critical challenge in collaboration concerns the distribution of roles between pharmaceutical companies and statutory health insurers in the area of digital health offerings. Health insurers are increasingly providing their own apps and coaching programs. This raises the difficult question of which digital tools will be reimbursed in the future, those developed by pharmaceutical companies or those offered by the insurers themselves. The issue touches on questions of competitive fairness, neutrality, and the long-term integration of such tools into standard care.

And then there’s the fact that there are actually many providers. And by the way, what role will the health insurance companies play in this? Because they also have apps and coaches, and so on. Will they say in the future, ‘Just use ours. We already have one, we’re not going to pay for one, two, three, or four more, just use ours’? In other words, how that will be settled. (Rheumatologist, male)

The collection and storage of health data by pharmaceutical companies is viewed with a degree of skepticism, particularly in the context of digital health applications. Ethical concerns arise around who ultimately owns the sensitive data, especially regarding mental health, and how that data is used. From the perspective of some participants, it is problematic when pharmaceutical companies not only provide digital tools but also gain access to large amounts of continuously collected health data. Trust in such offerings may be lower compared to similar products developed by professional societies or patient organizations.

I can also bring in a bit of an ethical aspect here. I think X mentioned earlier who the data is going to. So, is it really perhaps something to be critical of if pharmaceutical companies are producing DiGAs and then, let’s say, they hold very valuable data about health status, especially mental health, with a lot of continuous data in their hands? I mean, if I were a patient and there were two comparably good offerings, and one was developed by a professional society or maybe even a patient organization, I think I would feel more comfortable with that than if it came from a huge pharmaceutical company. But maybe I’m the only one who feels that way. (Rheumatologist, male)

The current distribution of tasks and responsibilities in managing health data within outpatient rheumatology is perceived as unclear and challenging. Looking ahead, there is a need to clarify the extent to which medical practices should and are allowed to handle the data collected through digital applications, and how involved different members of the practice team should be. Questions remain about who is responsible for which aspects of data monitoring and patient support, and what role medical assistants are expected to play. It is still uncertain whether they are meant to relieve physicians of certain tasks and how these responsibilities should be structured in everyday practice.

What I still see as a problem for myself is how we are going to monitor all of this at the end of the day, meaning to what extent we are still involved. So, the patient does something with it, the patient takes action. But what is ultimately our role in the practices? And where does the responsibility lie in terms of what things need to be taken care of, so to speak… How much should I, as a medical assistant, be involved? Do I need to be involved? Am I taking certain tasks off the doctor’s plate when working with patients? And there are still so many uncertainties for me regarding this. (Rheumatology medical assistant, female)

At the same time, the role and task of apps in patient care are seen as critical:

Of course, this is also an issue when we delegate control to health applications or health apps and then no longer know what kind of feedback the patients are receiving. Eventually, they come to us and say, ‘Well, I heard this and that from the app, so it must be this way.’ That’s actually a good point about how we can continue to monitor this and how much information we actually want to receive, so we are not overwhelmed by the information. Yes, that’s true. This probably needs to be regulated as well. (Internist, female)

At the same time, the role and function of health apps in patient care are viewed critically. Concerns arise especially when apps provide feedback directly to patients without physicians or medical staff being informed. This can lead to misunderstandings, confusion, and a loss of control over the treatment process. It also raises the question of how much information practices are realistically able and willing to process without becoming overwhelmed. The need for clear regulations and defined responsibilities in handling app-based patient feedback is becoming increasingly evident.

I believe that most of the available health apps, for example, are very disease-specific and therefore only partially able to digitally represent the true patient journey. When a patient has comorbidities, these apps or digital solutions quickly reach their limits. As was mentioned multiple times in the group, there are so many solutions available that it’s easy to lose track. I would like to express the wish for a collaboration between pharma and digital players to truly represent a complete digital journey that is not only disease-specific but patient-specific, capturing the full journey a patient might experience, including comorbidities and coexisting conditions that can emerge along the way. (Employee of a start-up, male)

There is also concern that physicians may be influenced in their prescribing decisions by digital offerings and email communications from the pharmaceutical industry. From a marketing perspective, digital tools may not serve solely as neutral sources of information, but also as instruments to promote specific products. The concern is that additional digital services could be strategically used to build patient trust or subtly steer treatment choices. This becomes particularly problematic when multiple providers operate simultaneously, making it difficult to clearly assess the content and underlying interests.

Because digital tools, especially when considered from a marketing perspective, seem to serve somewhat as a sales instrument, at least in my perception. By adding something digital, it incentivizes doctors to prescribe certain medications or opens digital channels to build trust with patients. This could become a problem when 15 different players are doing this. It becomes hard to discern what is true and what is not, and there is no real differentiation between these providers. (Employee of a start-up, patient with rheumatic disease, male)

Another point of criticism is the flood of information that physicians face as a result of digitalization. With the rise of digital offerings, particularly through email, many feel a constant pressure to be available and to respond. Whereas personal contact was previously easier to manage or decline, digital communication often creates a subtle expectation to engage. This development is perceived by some as burdensome and may lead to important content being overlooked or deliberately ignored, even if it might be relevant.

That’s a bit what I meant earlier. I hear it from my supervisors that they say, “Ever since everything went digital, I get so many emails and so many things that I don’t always necessarily want.” And that was what I initially meant. There used to be times when, if I didn’t have time, I could just say “not today,” and that was the end of it. But when I get it by email and there’s an expectation for a response, I feel more pressured. (Rheumatology medical assistant, female)

For a detailed overview of the category system, please refer to supplementary material 4.

## Discussion

### Principal results

The results of this focus group study highlight a central area of tension in the digital transformation. The pharmaceutical industry faces significant challenges and opportunities amid digital transformation. As a key stakeholder, it plays a central role in shaping communication strategies that impact patients, healthcare professionals, and the entire continuum of care. How companies adapt to these changes influences information dissemination and innovation within healthcare. While novel digital communication formats create new opportunities for contact, personal interactions are increasingly being pushed into the background. Rheumatology care providers perceive this constellation ambivalently. Although the intensity of digital contact is increasing, direct proximity to patients is being restricted on several levels. This raises the question of whether digitalization truly represents an improvement in the quality of participation or merely a quantitative “increase in contacts” [13, 14]. This ambivalence is supported by findings from Entezarjou et al. [15], who report that digital communication in healthcare is generally perceived as helpful, especially when it is associated with continuity of care. At the same time, they point to increased cognitive load, misunderstandings due to missing non-verbal cues, and a fragmentation of communication. Similarly, participants in our focus groups described feeling overwhelmed by the multitude of digital communication channels, which was perceived more as a quantitative increase than a qualitative improvement.

The results also reveal a shift in power dynamics. Some participants expressed a sense of dependency on the digital communication strategies of the pharmaceutical industry, which resembles the kind of asymmetrical relationship they previously observed in their own interactions with patients. This reflects a structural asymmetry: while digital tools open up new channels of communication, they also act as subtle mechanisms of control [16, 17]. Larkin et al. [18] similarly show that while pharmaceutical information services are considered useful by physicians, they are also suspected of exerting potential sometimes unconscious, influence on prescribing behavior. This perception resonates with our interviewees’ descriptions of a “digital oversupply” and a sense of being subtly steered.

Digital information overload is increasingly experienced as a challenge, or even as a burden. Physicians reported being affected by digital spam, floods of emails, and a loss of control over the ability to filter or reject communication impulses. Digital accessibility brings not only efficiency but also additional stress [19]. These issues are confirmed by current research: Butnaru et al. [20] report that general practitioners and medical staff working under high patient loads are particularly affected by so-called “techno-stress,” triggered by overload through emails, messaging services, and constant digital availability. Sbaffi et al. [21] likewise describe in a study among emergency physicians that an “always-on” culture, multidisciplinary communication pathways, and a constant stream of information can lead to guideline fatigue, stress, and impaired decision-making.

Particularly striking is the observation by several physicians that digital communication with the pharmaceutical industry is increasingly structured in a way that mirrors how they themselves interact digitally with patients, only in reverse. While pharmaceutical companies contact medical professionals through digital means, e.g., standardized emails, automated invitation systems, or webinars, physicians experience this as a one-way, distant transfer of information without real dialogue. At the same time, several participants noted that their own communication with patients has also increasingly taken on this character: low-contact, temporally fragmented, and technically mediated. This “double mirroring” suggests that structural communication patterns are reproduced along the care chain, often without participants being fully aware of these reciprocal dynamics. What is intended as a gain in efficiency often results in a relational decoupling: the mode of exchange changes more fundamentally than the content. Turkle [22] describes how technology reconfigures relationships without this transformation being actively reflected upon. We believe we are more connected through digital means, yet we are often more emotionally distant. Topol [23] also emphasizes that technological change not only shifts roles, for instance, from a consulting to a data-analyzing physician, but also leads to a new mode of communication that is shaped more by control than by relationship. Against this backdrop, it becomes clear that digital communication does not merely convey information but also implies and potentially reproduces particular relational patterns. The way physicians are treated may influence how they, in turn, interact with their patients. Thus, what is at stake is not merely the digitalization of individual touchpoints, but a broader question: how digital structures affect relationships, trust, and mutual responsibility within the healthcare system.

Another central theme concerns data management and the distribution of responsibility. The need for clearer role definitions mirrors participants’ uncertainty about who should monitor app-generated feedback, how responsibilities should be distributed within practices, and how much digital information healthcare teams can realistically process. The focus group discussions reveal a lack of clear regulation regarding who is responsible for handling sensitive health data and in what form. Physicians also expressed uncertainty about their role in monitoring digital applications. There is a clear need for transparent governance models [24, 25] to delineate responsibilities between practices, pharmaceutical companies, health insurers, and patients. This uncertainty about one’s role in digital data management is also reflected in current research. Silven et al. [26] report a widespread lack of clarity among physicians regarding their responsibilities in the use of digital health applications and call for professional organizations to take a stronger role in defining medical responsibilities. Meier et al. [27] similarly show that digital technologies can lead to shifts in moral responsibility, through so-called “responsibility gaps” in AI-based tools, but also through increased expectations placed on patients for self-management. A qualitative systematic review [28] highlights the need for collective governance structures that include political, medical, and digital stakeholders alike. These findings underscore the desire, voiced in our focus groups, for clear, cross-sectoral regulations in handling sensitive health data. Furthermore, governance should not only define individual responsibilities, but also foster collective accountability through joint committees, feedback loops, and participatory decision-making mechanisms across sectors.

At the same time, digital applications also offer significant innovative potential. Particularly emphasized were the opportunities they offer for practice-oriented study formats and personalized care enabled by real-time data from everyday clinical practice. However, this requires cooperative collaboration between stakeholders, without establishing parallel structures or conflicting goals [29, 30]. Recent studies also emphasize the potential of digital technologies to foster interdisciplinary collaboration and improve care, particularly in the field of rheumatology. Electronic health records, mobile applications, and AI-based tools can improve access to care, optimize disease management, and enhance research [31]. These technologies may reduce medical errors, improve doctor-patient communication, and support clinical decision-making [32]. Social media platforms such as Twitter have also become important sources of information and discussion within the medical community [33]. Recent qualitative studies in German-speaking countries confirm these insights: for example, Boy et al. [34] show that while digital applications, such as back pain-related DiGAs in rheumatology, are generally perceived as helpful, they are also associated with challenges such as lack of guidance, time constraints, training needs, and unclear reimbursement structures. Another study emphasizes that the integration of digital tools into existing practice workflows, along with a strong focus on usability and interdisciplinary connectivity, is key to sustainable implementation [35]. At the same time, studies highlight that challenges such as data protection concerns, limited digital literacy, and cultural differences between professional groups must be addressed [31, 36]. The successful implementation of digital technologies therefore requires strong social relationships and appropriate organizational structures [36]. Overall, digital applications have the potential to empower both patients and healthcare professionals, provided they are meaningfully embedded in interprofessional contexts.

### Practical implications

The digital transformation not only changes how physicians communicate with the pharmaceutical industry, but also reshapes understandings of roles, responsibilities, and ethical positioning. These implications are grounded in the themes identified in our data—particularly (i) perceived information overload and reduced dialogical exchange, (ii) blurred role boundaries in data monitoring, and (iii) concerns about commercial influence and data governance. For example, participants described a “digital overload” and the feeling of being kept engaged through frequent digital contact (Rheumatologist, male), while practice staff highlighted uncertainties regarding responsibilities for monitoring and responding to app-generated information (Rheumatology medical assistant, female). Building on these themes, the following implications emphasize governance, accountability, and patient-centered integration [24, 25].A key area for action is the establishment of trust in the digital space, particularly concerning the handling of health data by commercial providers. This aligns with participants’ concerns about data ownership and trust when commercial actors provide digital tools and collect sensitive health data, which several interviewees described as ethically problematic and potentially undermining confidence in digital care solutions. As both our interviews and existing studies indicate, many physicians express considerable skepticism toward the pharmaceutical industry when it comes to data protection, data sovereignty, and secondary use of data [37, 38]. Addressing this mistrust requires transparent communication structures, accountability mechanisms, and opportunities for participation for both medical professionals and patients [16].

Moreover, the fragmentation of digital offerings must be overcome. Many applications are disease-specific and not suited to reflect complex or multimorbid care pathways. A patient-centered, integrated care approach instead requires cross-sector collaboration that consciously transcends existing boundaries of responsibility [39, 40].

Adherence and long-term sustainability of digital applications also remain a challenge: studies show that while many health apps are used initially, they are rarely integrated into care in the long-term [41, 42]. Contributing factors include limited practicality, technical instability, lack of integration into practice routines, and rapid technological obsolescence that regulatory processes often fail to keep up with [37]. Closer alignment with everyday clinical practice and continuous development are therefore essential.

Structural barriers to digital participation must also be addressed more proactively. Digital literacy is unequally distributed, both among patients and healthcare professionals. Differences in digital infrastructure between urban and rural regions also contribute to unequal access to digital health in rheumatology care [43, 44]. This dimension of the “digital divide” is widely recognized in the literature as a major barrier to equitable digitalization in healthcare [45].

Finally, to manage the multitude of digital communication stimuli effectively and avoid overload, systematic information management and targeted eHealth literacy training are needed [19, 45]. Only then can digital transformation be harnessed as an opportunity to improve care, communication, and collaboration, rather than becoming a burden.

### Limitations

This study has several limitations that should be considered when interpreting its findings. While the qualitative design allowed for in-depth exploration of stakeholder perspectives, the sample size and scope were limited. Although we aimed for heterogeneity, all participating rheumatologists were male, which may limit the diversity of perspectives captured and may have influenced how digital industry interactions were framed (e.g., professional norms, communication styles, and perceived role expectations). Transferability to female rheumatologists and more gender-diverse rheumatology teams should therefore be made with caution. Participation may also have been influenced by self-selection bias. Individuals with a particular interest in digital health or previous experience in interacting with the pharmaceutical industry may have been more inclined to take part, potentially shaping the range of attitudes reflected in the data. The use of online focus groups was methodologically appropriate given the topic of digital transformation. However, this format may have constrained elements of group interaction, such as nonverbal cues or informal exchanges, possibly affecting the richness of certain discussions. Moreover, the study centres on the perspectives of healthcare stakeholders and does not systematically include views from patients or pharmaceutical industry representatives. Future studies should incorporate these additional voices to better capture the complexity of digital relationships and role expectations.

## Conclusion

This study shows that the digital transformation not only gives rise to new forms of interaction between physicians and the pharmaceutical industry but also entails fundamental changes in the perception of roles, responsibilities, and relationships of trust. The stakeholders interviewed perceive digital communication formats as ambivalent: while they enable greater efficiency and new opportunities for collaboration, they are also experienced as distant, overwhelming, and potentially controlling.

It becomes particularly clear that digitalization is not merely a technical issue, but above all a social and ethical challenge. Questions related to data management, the distribution of responsibilities, the fragmentation of services, and inclusive participation call for clear governance structures and transparent, participatory negotiation processes. These are essential to strengthen trust in digital collaboration and to fully realize its potential for high-quality care.

To shape future-oriented digital interactions between the healthcare sector and industry, structural integration, cultural sensitivity, and a critical awareness of power dynamics and communication processes are just as crucial as technological innovation. Digital transformation in healthcare should therefore be understood as a malleable, reflective process, not merely as technological progress, but as a social renegotiation of relationships, responsibility, and care.

## Supplementary Information

Below is the link to the electronic supplementary material.

Supplementary Material 1

Supplementary Material 2

Supplementary Material 3

Supplementary Material 4

## Data Availability

All data supporting the findings of this study are available within the paper and its additional files. The data sets are available on reasonable request from the corresponding author.

## Abbreviations

DiGA: Digital Health Application (German: Digitale Gesundheitsanwendung)
COREQ: Consolidated Criteria for Reporting Qualitative Research
RFA: Rheumatology Medical Assistant
MAXQDA: MAX Qualitative Data Analysis (software for qualitative data analysis)
