# Supplementary material for: Digital interactions with the pharmaceutical industry: a qualitative focus group study on the perspectives of rheumatology care providers in Germany
Source: BMC Rheumatol. 2026 Feb 11;10:23. doi: 10.1186/s41927-026-00623-1 (PMC12998326; doi:10.1186/s41927-026-00623-1)
Supplement: Supplementary file 1 — Supplementary Material 1 [file 41927_2026_623_MOESM1_ESM.pdf]

Supplementary Material 1
Interview Guide

<table>
<tr><td>Guiding question/ narrative impulse</td><td>Check aspects</td><td>Concretizing questions</td><td>Maintenance and control questions</td></tr>
<tr><td colspan="4">Introduction: Open introductory question

What impact is digitalization having on your collaboration with the pharmaceutical industry?</td></tr>
<tr><td>How has personal contact with pharmaceutical companies changed through digital channels?</td><td>Communication & Interaction</td><td>What digital forms of communication (e.g., webinars, social media, emails) are being used – and how are they perceived?

Is there a sense of digital overload (“spam feeling”)?</td><td rowspan="5">Can you tell more about this?

And then?

What was that like for you?

How do you see it?

Can you elaborate on that, please?

Could you give an example of that, please?

What do you mean specifically?</td></tr>
<tr><td>What role do digital tools (e.g., ePROs, sensors, apps) play in clinical trials?</td><td>Research & Clinical Studies</td><td>How are Big Data/AI being used in research?

What expectations exist regarding real-world data and its quality?</td></tr>
<tr><td>What experiences have you had with DiGAs in everyday clinical practice?</td><td>Digital Health Applications (DiGAs)</td><td>To what extent are pharmaceutical companies involved in digital therapies?

What is the attitude towards pharma-developed DiGAs versus independent solutions?</td></tr>
<tr><td>What role do patients play in the digital transformation?</td><td>Patient Involvement & Real-World Care</td><td>How well is patient feedback from digital tools integrated into everyday clinical routines?

What challenges arise from the “information overload” on the patient side?</td></tr>
<tr><td>How are data privacy, conflicts of interest, and</td><td>Ethical & Regulatory Aspects</td><td>Who monitors the content and data flows from digital offerings?</td></tr>
</table>

transparency being handled?		What might meaningful governance look like?	
What potential do you see in digital collaboration with pharmaceutical companies – particularly for care, research, and innovation?	**Outlook & Opportunities**	What developments seem desirable or likely?<br><br>Where is political or regulatory action needed?<br><br>What role should professional societies, patient organizations, or health insurers play?	
