# Supplementary material for: Digital interactions with the pharmaceutical industry: a qualitative focus group study on the perspectives of rheumatology care providers in Germany
Source: BMC Rheumatol. 2026 Feb 11;10:23. doi: 10.1186/s41927-026-00623-1 (PMC12998326; doi:10.1186/s41927-026-00623-1)
Supplement: Supplementary file 2 — Supplementary Material 2 [file 41927_2026_623_MOESM2_ESM.pdf]

Supplementary Material File 2

Consolidated criteria for reporting qualitative studies (COREQ): 32-item checklist

Developed from:
Tong A, Sainsbury P, Craig J. Consolidated criteria for reporting qualitative research (COREQ): a 32-item checklist for interviews and focus groups. *International Journal for Quality in Health Care*. 2007. Volume 19, Number 6: pp. 349 – 357

Topic	Item No.	Guide questions/description	Reported on Page #
Domain 1: Research team and reflexivity			
Personal Characteristics			
Interviewer/facilitator	1	Which author/s conducted the interview or focus group?	Author information
Credentials	2	What were the researcher's credentials? E.g. PhD, MD	Author information
Occupation	3	What was their occupation at the time of the study?	Author information
Gender	4	Was the researcher male or female?	Author information
Experience and training	5	What experience or training did the researcher have?	N/A
Relationship with participants			
Relationship established	6	Was a relationship established prior to study commencement?	5
Participant knowledge of the interviewer	7	What did the participants know about the researcher? e.g. personal goals, reasons for doing the research	5
Interviewer characteristics	8	What characteristics were reported about the inter viewer/facilitator? e.g. Bias, assumptions, reasons and interests in the research topic	5
Domain 2: study design			
Theoretical framework			
Methodological orientation and Theory	9	What methodological orientation was stated to underpin the study? e.g. grounded theory, discourse analysis, ethnography, phenomenology, content analysis	5
Participant selection			
Sampling	10	How were participants selected? e.g. purposive, convenience, consecutive, snowball	5
Method of approach	11	How were participants approached? e.g. face-to-face,	5

		telephone, mail, email	
Sample size	12	How many participants were in the study?	7
Non-participation	13	How many people refused to participate or dropped out? Reasons?	N/A
Setting			
Setting of data collection	14	Where was the data collected? e.g. home, clinic, workplace	5
Presence of non-participants	15	Was anyone else present besides the participants and researchers?	N/A
Description of sample	16	What are the important characteristics of the sample? e.g. demographic data, date	7
Data collection			
Interview guide	17	Were questions, prompts, guides provided by the authors? Was it pilot tested?	5
Repeat interviews	18	Were repeat interviews carried out? If yes, how many?	N/A
Audio/visual recording	19	Did the research use audio or visual recording to collect the data?	5
Field notes	20	Were field notes made during and/or after the interview or focus group?	5
Duration	21	What was the duration of the interviews or focus group?	6
Data saturation	22	Was data saturation discussed?	5
Transcripts returned	23	Were transcripts returned to participants for comment and/or correction?	N/A
Domain 3: analysis and findings			
Data analysis			
Number of data coders	24	How many data coders coded the data?	5
Description of the coding tree	25	Did authors provide a description of the coding tree?	Supplementary Material 3<br>Supplementary Material 4
Derivation of themes	26	Were themes identified in advance or derived from the data?	5
Software	27	What software, if applicable, was used to manage the data?	5
Participant checking	28	Did participants provide feedback on the findings?	N/A
Reporting			
Quotations presented	29	Were participant quotations presented to illustrate the themes/findings? Was each quotation identified? e.g. participant number	7-14
Data and findings consistent	30	Was there consistency between the data presented and the	7-18

		findings?	
Clarity of major themes	31	Were major themes clearly presented in the findings?	7-18
Clarity of minor themes	32	Is there a description of diverse cases or discussion of minor themes?	7-18
