# Supplementary material for: Digital interactions with the pharmaceutical industry: a qualitative focus group study on the perspectives of rheumatology care providers in Germany
Source: BMC Rheumatol. 2026 Feb 11;10:23. doi: 10.1186/s41927-026-00623-1 (PMC12998326; doi:10.1186/s41927-026-00623-1)
Supplement: Supplementary file 3 — Supplementary Material 3 [file 41927_2026_623_MOESM3_ESM.pdf]

Category system: Impact of Digitalization on Collaboration with the Pharmaceutical Industry in Rheumatology

Category	Definition	Anchor quote
Shift in Communication Channels	Change in communication practices through social media and direct digital interaction with patients.	*"Over the past three to four years, I have noticed increasing efforts in communication, with new communication channels emerging that did not previously exist or that I, as an affected individual, had not registered or perceived at the time."* (Digital health entrepreneur, male)
Digital Transformation in Physician-Pharma Communication	Decline in personal contact and increase in digital formats in the interaction between practices and the pharmaceutical industry.	*"Due to digitalization and the significantly reduced direct contact with the pharmaceutical industry, our interactions have become more distanced. They now provide us with more information digitally and are less physically present."* (Rheumatologist, male)
Integration of Digital Endpoints in Clinical Research	Incorporation of digital measures into study designs, enhancing collaboration.	*"Yes, there are increasingly more digital endpoints, or rather an additional Clinical Outcome Assessment, facilitated through apps and similar technologies, which are now being integrated into clinical studies. In this sense, clinical studies have also evolved, with more digital endpoints being available on the market, validated, and incorporated into research. The pharmaceutical industry is, of course, examining whether we have algorithms to predict treatment responses, which we do not yet have, at least not properly validated."* (Rheumatologist, male)
Increase in Digital Educational Offerings	Expansion of digital training modules by the pharmaceutical industry, leading to broader reach and improved accessibility.	*"Essentially, it is an addition; the use of digital training modules provided by the industry has significantly increased. For example, in-person events and other (unintelligible) events have risen exponentially, while the number of participants has rather decreased. However, accessibility and reach have increased significantly."* (Rheumatologist, male)
Higher Communication	More frequent exchanges via digital formats, even	*"Yes, that's exactly what I meant with the lectures and offerings from the*

Frequency Despite Physical Distance	though in-person meetings are less common.	*pharmaceutical company. I also have the feeling that, although we don't see the pharmaceutical company in person, we actually have more contact with them because we are more frequently invited to online meetings or asked to speak at various events."* (Internist, female)
Stronger Networking with Digital Health Startups	Simplified communication and growing interest by the pharmaceutical industry digital therapies.	*"We come from the digital therapies field, and we've noticed that the pharmaceutical industry is closely monitoring this. They are essentially observing whether the whole topic of DiGAs and digital therapies is moving out of the proof-of-concept phase and really generating evidence and added value, including pharmacoeconomic benefits. It's slowly gaining momentum. Also, the discussions we have with pharma are becoming much easier because they are now more aware of what they actually want to achieve."* (Employee of a start-up, male)
Increased Efficiency Through Digital Tools	Use of video conferencing tools like Zoom enables faster and more targeted communication.	*"Overall, I personally find communication with pharmaceutical companies to be more targeted and easier due to digitalization, because you can just quickly talk via Zoom now. You no longer need to arrange on-site appointments... I think it has become more efficient and easier. So, I view this rather positively."* (Rheumatologist, male)
Mirrored Communication Structures	Parallels between how physicians interact with patients and how the pharmaceutical industry interacts with physicians.	*"I think if you ask us doctors, we always believe we're at the center, that we're in control and determine the pace and nature of digitalization. But if we're being honest and talk about the pharmaceutical industry, they're essentially playing the same game with us that we play with patients. Meaning, we're distancing ourselves from patients, trying to provide them with lots of information, seeing them less frequently because things are becoming more digital. And the pharmaceutical industry is doing*

		exactly the same with us. We see them less often in person. In the end, we're also a kind of pawn,they keep us engaged with a sort of digital overload. To be honest, the same thing we're doing with patients is what the pharmaceutical industry is doing with us." (Rheumatologist, male)
Pharmaceutical Industry as Pragmatic Project Partner	Industry perceived as a potential collaborator with the resources and pragmatism to advance projects.	*"Well, I think when you look at projects nowadays, funding is often a major issue. In that regard, I definitely see the pharmaceutical industry as a potential partner,at the very least, they have the resources to implement and push certain topics forward. They may also take a somewhat more pragmatic approach than what we typically see in the statutory health insurance environment or in academic research contexts."* (Employee of a start-up, patient with rheumatic disease, male)
Potential for Practice-Based Real World Data Collection	Use of digital infrastructure to collect and utilize everyday care data beyond traditional clinical trials.	*"I believe that collaboration with the pharmaceutical industry especially for office-based physicians offers tremendous opportunities, particularly when care is conceived in a digital context. Assuming, and this is a hypothesis, that the treating physician remains the central point of contact for the patient, while a digital connection is maintained, then the treating physician, or rheumatologist in this case, would be in a significantly better starting position. Specialists treating chronic conditions could, more or less, maintain their own practice-based registry with tightly monitored data streams that are collected automatically, without the need for active manual input. The major opportunity arising from this is that we will, for the first time, have large datasets from patients' everyday lives, outside the scope of clinical trials. This enables truly personalized adjustments and allows observational studies and similar research to be organized more broadly and*

		decentrally in daily practice. Different networks of practices could initiate various studies without much effort." (Employee of a start-up, male)
