# Supplementary material for: Digital interactions with the pharmaceutical industry: a qualitative focus group study on the perspectives of rheumatology care providers in Germany
Source: BMC Rheumatol. 2026 Feb 11;10:23. doi: 10.1186/s41927-026-00623-1 (PMC12998326; doi:10.1186/s41927-026-00623-1)
Supplement: Supplementary file 4 — Supplementary Material 4 [file 41927_2026_623_MOESM4_ESM.pdf]

Category system: Challenges in Collaboration with the Pharmaceutical Industry

Category	Definition	Anchor quote
Information Overload from Digital Communication	The high frequency of digital contact attempts, especially via email, leads to overload and a sense of pressure among physicians.	*"Exactly, what comes to mind spontaneously with these inputs is the offer of many digital tools that pharmaceutical companies provide to patients. I think as a patient, you'd probably lose track of them pretty quickly."* (Internist, female)
Lack of Clarity in Digital Patient Tools	The abundance of digital tools and apps, often provided by pharmaceutical companies, can overwhelm patients.	*"And then there's the fact that there are actually many providers. And by the way, what role will the health insurance companies play in this? Because they also have apps and coaches, and so on. Will they say in the future, 'Just use ours. We already have one, we're not going to pay for one, two, three, or four more, just use ours'? In other words, how that will be settled."* (Rheumatologist, male)
Competition Between Health Insurers and Industry	Uncertainty about whether future reimbursement will cover digital health tools from insurers or those developed by pharmaceutical companies.	*"I can also bring in a bit of an ethical aspect here. I think X mentioned earlier who the data is going to. So, is it really perhaps something to be critical of if pharmaceutical companies are producing DiGAs and then, let's say, they hold very valuable data about health status, especially mental health, with a lot of continuous data in their hands? I mean, if I were a patient and there were two comparably good offerings, and one was developed by a professional society or maybe even a patient organization, I think I would feel more comfortable with that than if it came from a huge pharmaceutical company. But maybe I'm the only one who feels that way."* (Rheumatologist, male)
Criticism of Data Collection by Pharmaceutical Companies	Concerns regarding data ownership and ethical issues when pharma companies collect health data via their own digital tools.	*"What I still see as a problem for myself is how we are going to monitor all of this at the end of the day, meaning to what extent we are still involved. So, the patient does something with it, the patient takes action. But what is ultimately our role in the practices? And where does the responsibility lie in terms of what things need to be taken care of, so to speak… How much should I, as a medical assistant, be involved? Do I need*

		to be involved? Am I taking certain tasks off the doctor's plate when working with patients? And there are still so many uncertainties for me regarding this." (Rheumatology medical assistant, female)
Unclear Roles in Practice-Based Data Management	Lack of defined responsibilities regarding who in the medical practice handles and monitors patient data generated by digital tools.	*"Of course, this is also an issue when we delegate control to health applications or health apps and then no longer know what kind of feedback the patients are receiving. Eventually, they come to us and say, 'Well, I heard this and that from the app, so it must be this way.' That's actually a good point about how we can continue to monitor this and how much information we actually want to receive, so we are not overwhelmed by the information. Yes, that's true. This probably needs to be regulated as well."* (Internist, female)
Critical View of App Roles in Patient Care	Concerns about loss of control and confusion caused by apps that provide feedback to patients without informing healthcare providers.	*"I believe that most of the available health apps, for example, are very disease-specific and therefore only partially able to digitally represent the true patient journey. When a patient has comorbidities, these apps or digital solutions quickly reach their limits. As was mentioned multiple times in the group, there are so many solutions available that it's easy to lose track. I would like to express the wish for a collaboration between pharma and digital players to truly represent a complete digital journey that is not only disease-specific but patient-specific, capturing the full journey a patient might experience, including comorbidities and coexisting conditions that can emerge along the way."* (Employee of a start-up, male)
Potential Influence on Medical Decision Making	The concern that digital offerings from pharmaceutical companies may subtly influence prescribing behavior.	*"Because digital tools, especially when considered from a marketing perspective, seem to serve somewhat as a sales instrument, at least in my perception. By adding something digital, it incentivizes doctors to prescribe certain medications or opens digital channels to build trust with patients. This could become a problem when 15 different players are doing this. It becomes hard to discern what is true and what is not, and there is no real*

		differentiation between these providers." (Employee of a start-up, patient with rheumatic disease, male)
Information Overload and Expectation Pressure	The increasing volume of digital communication, particularly via email, creates a sense of constant availability and subtle pressure to respond.	*"That's a bit what I meant earlier. I hear it from my supervisors that they say, "Ever since everything went digital, I get so many emails and so many things that I don't always necessarily want." And that was what I initially meant. There used to be times when, if I didn't have time, I could just say "not today," and that was the end of it. But when I get it by email and there's an expectation for a response, I feel more pressured."* (Rheumatology medical assistant, female)
